# Supplementary material for: Analyzing the Therapeutic Efficacy of Bis-Choline-Tetrathiomolybdate in the Atp7b−/− Copper Overload Mouse Model
Source: Biomedicines. 2021 Dec 8;9(12):1861. doi: 10.3390/biomedicines9121861 (PMC8698685; doi:10.3390/biomedicines9121861)
Supplement: Supplementary file 1 [file biomedicines-09-01861-s001.zip › biomedicines-1472356-supplementary.pdf]

biomedicines

MDPI

Supplements

Analyzing the Therapeutic Efficacy of Bis-Choline-Tetrathiomolybdate in the *Atp7b*$^{-/-}$ Copper Overload Mouse Model

Philipp Kim [1,†], Chengcheng Christine Zhang [2,†], Sven Thoröe-Boveleth [3], Eva Miriam Buhl [4], Sabine Weiskirchen [1], Wolfgang Stremmel [5], Uta Merle [2,*] and Ralf Weiskirchen [1,*]

Supplementary Tables

Table S1. Summary of animal treatments used in dose finding and therapeutic efficiency experiment

A) Dose finding experiment

Cons. no.	Genotype	Age at start (wks)	Age at end (wks)	Treatment
1-3	WT	9	9	none
4-6	WT	9	13	100 µl water
7-9	*Atp7b*$^{-/-}$	9	13	1 mg bc-TTM/kg bw
10-12	*Atp7b*$^{-/-}$	9	13	5 mg bc-TTM/kg bw
13-15	*Atp7b*$^{-/-}$	9	13	10 mg bc-TTM/kg bw

B) Therapeutic efficacy experiment

Cons. no.	Genotype	Age at start (wks)	Age at end (wks)	Treatment
16-20	WT	36	36	none
21-25	*Atp7b*$^{-/-}$	36	36	none
26-30	*Atp7b*$^{-/-}$	36	44	100 µl water
31-35	*Atp7b*$^{-/-}$	36	44	1 mg bc-TTM/kg bw
36-40	*Atp7b*$^{-/-}$	36	44	5 mg bc-TTM/kg bw
41-46	*Atp7b*$^{-/-}$	36	44	200 mg TETA/kg bw
47-50	*Atp7b*$^{-/-}$	36	44	200 mg DPA/kg bw

Comments: Animals received oral gavages of indicated quantities of bis-choline tetrathiomolybdate (bc-TTM), trientine (TETA), or D-penicillamine (DPA) dissolved in 100 µl water four times a week per oral gavage for 4 (dose finding experiment) or 8 consecutive weeks (therapeutic efficacy experiment). Animals receiving water only or sacrificed without treatment served as controls. Abbreviations used are: *Atp7b*$^{-/-}$, mouse deficient for the *Atp7b* gene; bw, body weight; wks, weeks; WT, wild type.

Table S2. Summary of all animals used in dose finding experiment and therapeutic efficiency experiment

A) Dose finding experiment

Animal						Serum (ng/mL)					Liver (ng/g)				
**Cons. no.**	**Inter. no.**	**Genotype**	**Age at start (wks)**	**Age at end (wks)**	**Treatment**	**^{63}Cu**	**^{65}Cu**	**Fe**	**Mo**	**Zn**	**^{63}Cu**	**^{65}Cu**	**Fe**	**Mo**	**Zn**
1	1394	WT	9	9	none	628	270	9380	<20	951	3172	1347	158024	862	27603
2	1395	WT	9	9	none	550	237	4520	<20	802	2889	1209	149471	865	28519
3	1396	WT	9	9	none	506	221	6880	<20	755	3193	1352	96619	903	27236
4	1334	WT	9	13	100 µl water	538 (h)	232	24900	<20	1260	3306	1380	121569	999	30444
5	1335	WT	9	13	100 µl water	582	249	11200	<20	1100	3143	1326	97426	919	28296
6	1336	WT	9	13	100 µl water	563	244	6990	<20	893	2876	1208	109373	939	29313
7	729	*Atp7b$^{-/-}$*	9	13	1 mg bc-TTM/kg bw	877	379	3030	36.7	1200	120496	52493	190884	899	45838
8	730	*Atp7b$^{-/-}$*	9	13	1 mg bc-TTM/kg bw	846	361	1940	110	1040	162165	69555	350510	1503	53080
9	731	*Atp7b$^{-/-}$*	9	13	1 mg bc-TTM/kg bw	1170	502	3880	171	1440	118231	48637	180707	1402	47877
10	735	*Atp7b$^{-/-}$*	9	13	5 mg bc-TTM/kg bw	900	388	4820	596	994	96124	43313	135258	6343	47482
11	736	*Atp7b$^{-/-}$*	9	13	5 mg bc-TTM/kg bw	1250	537	7890	952	1110	138665	50567	180936	12232	57085
12	737	*Atp7b$^{-/-}$*	9	13	5 mg bc-TTM/kg bw	1130	487	6570	780	1120	93806	41088	120552	11497	51134
13	747	*Atp7b$^{-/-}$*	9	13	10 mg bc-TTM/kg bw	781	335	2180	610	786	108647	49385	158444	14484	50079
14	748	*Atp7b$^{-/-}$*	9	13	10 mg bc-TTM/kg bw	1030	444	4160	736	824	108035	44057	153624	11637	55575
15	749	*Atp7b$^{-/-}$*	9	13	10 mg bc-TTM/kg bw	1360	589	4640	988	1100	124861	53232	156175	11025	64100

B) Therapeutic efficacy experiment

**Animal**						**Serum (ng/mL)**					**Liver (ng/g)**				
**Cons. no.**	**Inter. no.**	**Genotype**	**Age at start (wks)**	**Age at end (wks)**	**Treatment**	**^{63}Cu**	**^{65}Cu**	**Fe**	**Mo**	**Zn**	**^{63}Cu**	**^{65}Cu**	**Fe**	**Mo**	**Zn**
16	1312	WT	36	36	none	593	252	6290	<20	785	2791	1175	119143	872	25959
17	1313	WT	36	36	none	666	286	5900	<20	1020	4190	1795	193935	1084	34421
18	1314	WT	36	36	none	605	258	4220	<20	985	3369	1430	151696	993	30739
19	1315	WT	36	36	none	638	276	10000	<20	875	3098	1316	169657	871	27099
20	1316	WT	36	36	none	548	238	7420	<20	738	2633	1118	201784	814	24169
21	616	$Atp7b^{-/-}$	36	36	none	520	227	2270	<20	807	17209	7271	174644	475	23675
22	617	$Atp7b^{-/-}$	36	36	none	497	214	1460	<20	680	11798	5192	193061	562	22430
23	621	$Atp7b^{-/-}$	36	36	none	656	281	2470	<20	783	1430	604	194704	295	22901
24	622	$Atp7b^{-/-}$	36	36	none	460	196	2220	<20	766	6260	2643	201281	388	21929
25	624	$Atp7b^{-/-}$	36	36	none	594	252	2860	<20	672	11119	4806	197838	725	23350
26	608	$Atp7b^{-/-}$	36	44	100 µl water	543	236	4830	<20	884	4475	1885	267766	ND	16560
27	609	$Atp7b^{-/-}$	36	44	100 µl water	559	241	2990	<20	746	20096	8579	339431	900	34279
28	610	$Atp7b^{-/-}$	36	44	100 µl water	580	247	3190	<20	756	54770	23681	286852	901	42215
29	613	$Atp7b^{-/-}$	36	44	100 µl water	539	233	2820	<20	879	44918	20115	220293	896	41476
30	615	$Atp7b^{-/-}$	36	44	100 µl water	423	182	3000	<20	876	14177	6147	206958	603	26894
31	670	$Atp7b^{-/-}$	36	44	1 mg bc-TTM/kg bw	542	231	3340	59.1	878	15600	6700	284762	1183	28300
32	689	$Atp7b^{-/-}$	36	44	1 mg bc-TTM/kg bw	668	287	3340	52.9	719	10266	4420	204920	773	22634
33	690	$Atp7b^{-/-}$	36	44	1 mg bc-TTM/kg bw	663	286	3650	49.9	729	4417	1880	209585	450	16771
34	683	$Atp7b^{-/-}$	36	44	1 mg bc-TTM/kg bw	526	226	3620	66.1	649	9156	3963	223212	970	27059
35	684	$Atp7b^{-/-}$	36	44	1 mg bc-TTM/kg bw	629	270	2820	89.3	1100	33761	14469	248367	1558	40312
36	672	$Atp7b^{-/-}$	36	44	5 mg bc-TTM/kg bw	1180	506	3450	524	832	32968	14036	203980	7458	32160
37	677	$Atp7b^{-/-}$	36	44	5 mg bc-TTM/kg bw	935	399	1910	305	671	25273	10727	270482	5429	28941
38	678	$Atp7b^{-/-}$	36	44	5 mg bc-TTM/kg bw	890	385	2690	304	740	48144	22162	208095	4038	40382
39	693	$Atp7b^{-/-}$	36	44	5 mg bc-TTM/kg bw	857	362	3140	269	756	10246	4512	189731	4546	24936
40	697	$Atp7b^{-/-}$	36	44	5 mg bc-TTM/kg bw	847	359	3740	310	919	24186	10355	205382	4598	32985
41	627	$Atp7b^{-/-}$	36	44	200 mg TETA/kg bw	402	172	3190	<20	979	7985	3427	206194	442	21144
42	628	$Atp7b^{-/-}$	36	44	200 mg TETA/kg bw	404	174	5820	<20	1150	6520	2733	259989	777	29216
43	629	$Atp7b^{-/-}$	36	44	200 mg TETA/kg bw	436	187	2900	<20	1040	NN	NN	NN	NN	NN
44	630	$Atp7b^{-/-}$	36	44	200 mg TETA/kg bw	485	207	5270	<20	1090	24282	10489	355734	887	34592
45	637	$Atp7b^{-/-}$	36	44	200 mg TETA/kg bw	428	184	3770	<20	974	4291	1796	206836	752	28787
46	643	$Atp7b^{-/-}$	36	44	200 mg TETA/kg bw	516	221	9370	<20	978	2467	1024	181208	675	25058

47	661	*Atp7b*$^{-/-}$	36	44	200 mg DPA/kg bw	467	201	2450	<20	965	10219	4443	191585	522	24954
48	664	*Atp7b*$^{-/-}$	36	44	200 mg DPA/kg bw	311	134	2450	<20	880	3742	1578	207307	317	20720
49	665	*Atp7b*$^{-/-}$	36	44	200 mg DPA/kg bw	354	152	2240	<20	870	4186	1769	237961	803	26232
50	666	*Atp7b*$^{-/-}$	36	44	200 mg DPA/kg bw	388	166	2900	<20	837	NN	NN	NN	NN	NN
51	1317	WT	NA	44	none	720	311	5990	<20	1030	NN	NN	NN	NN	NN
52	1321	WT	NA	44	none	828	354	3970	<20	1160	NN	NN	NN	NN	NN
53	1322	WT	NA	44	none	632	273	8650	<20	926	NN	NN	NN	NN	NN
54	1323	WT	NA	44	none	651	278	3050	<20	785	NN	NN	NN	NN	NN
55	1327	WT	NA	44	none	750	323	9100	<20	1040	NN	NN	NN	NN	NN
56	705	*Atp7b*$^{-/-}$	36	44	200 mg DPA/kg bw	563	241	9960	<20	743	NN	NN	NN	NN	NN
57	706	*Atp7b*$^{-/-}$	36	44	200 mg DPA/kg bw	420	181	6000	<20	954	NN	NN	NN	NN	NN
58	788	*Atp7b*$^{-/-}$	NA	52	100 µl water	490	207	4110	<20	1130	NN	NN	NN	NN	NN
59	789	*Atp7b*$^{-/-}$	NA	52	100 µl water	318	136	2460	25.5	915	NN	NN	NN	NN	NN
60	790	*Atp7b*$^{-/-}$	NA	52	100 µl water	305	130	4420	<20	1220	NN	NN	NN	NN	NN
61	795	*Atp7b*$^{-/-}$	NA	9	none	280	123	11600	<20	1240	NN	NN	NN	NN	NN
62	796	*Atp7b*$^{-/-}$	NA	9	none	219	96.2	3160	<20	1060	NN	NN	NN	NN	NN
63	797	*Atp7b*$^{-/-}$	NA	9	none	215	91.5	3640	<20	1610	NN	NN	NN	NN	NN
64	947	*Atp7b*$^{-/-}$	NA	52	100 µl water	358	153	5370	<20	1070	130044	57754	169332	645	55785
65	952	*Atp7b*$^{-/-}$	NA	52	100 µl water	264	114	11500	<20	1300	130620	49320	136785	810	63878
66	953	*Atp7b*$^{-/-}$	NA	52	100 µl water	266	115	5480	<20	1090	125590	55905	101968	833	65213
67	966	*Atp7b*$^{-/-}$	NA	52	100 µl water	338	145	3420	<20	978	124825	53384	127965	765	61552
68	967	*Atp7b*$^{-/-}$	NA	52	100 µl water	400	173	4260	<20	997	130153	55292	159734	662	52425
69	968	*Atp7b*$^{-/-}$	NA	52	100 µl water	514	220	3780	<20	1050	210226	89983	196688	860	75087
70	969	*Atp7b*$^{-/-}$	NA	52	100 µl water	567	243	6890	<20	1200	128381	55395	162816	597	51311
71	970	*Atp7b*$^{-/-}$	NA	52	100 µl water	465	199	3720	<20	998	143405	60611	161232	683	58968
72	971	*Atp7b*$^{-/-}$	NA	52	100 µl water	369	156	3410	<20	1220	143570	60083	169769	724	58498

Comments: Animals received oral gavages of indicated quantities of bis-choline-tetrathiomolybdate (TTM), trientine (TETA), or D-penicillamine (DPA) dissolved in 100 µl water four times a week per oral gavage for 4 (dose finding experiment) or 8 consecutive weeks (therapeutic efficacy experiment). Animals receiving water only or sacrificed without treatment served as controls. Abbreviations used are: *Atp7b*$^{-/-}$, mouse deficient for the *Atp7b* gene; bw, body weight; ND, not detectable (below the lower limit of quantification); wks, weeks; WT, wild type.

Table S3. Concentrations of selected elements as determined by laser ablation inductively coupled mass spectrometry

Genotype	Treatment*	Age (wks) at beginning/end	n	Element concentration [μg/g liver tissue]**			
				^{56}Fe	^{55}Mn	^{64}Zn	^{63}Cu
WT	none	9/9	3	549.33 ± 57.11	1.07 ± 0.14	37.33 ± 2.88	3.41 ± 0.28
WT	100 μl water	9/13	3	449 ± 78.80	1.19 ± 0.05	39.7 ± 1.84	3.45 ± 0.30
Atp7b$^{-/-}$	none	20/20	5	314.2 ± 37.88	0.692 ± 0.10	43.16 ± 3.47	112.72 ± 13.26
Atp7b$^{-/-}$	1 mg bc-TTM/kg bw	9/13	3	998 ± 179.63	0.90 ± 0.14	78.27 ± 3.76	203.03 ± 21.91
Atp7b$^{-/-}$	5 mg bc-TTM/kg bw	9/13	3	468 ± 186.57	0.75 ± 0.34	49.37 ± 23.75	106.9 ± 45.65
Atp7b$^{-/-}$	10 mg bc-TTM/kg bw	9/13	3	334 ± 78.89	0.67 ± 0.11	41.93 ± 7.05	86.9 ± 15.69
WT	none	36/36	5	371 ± 96.44	0.58 ± 0.07	22.15 ± 3.86	2.09 ± 0.28
Atp7b$^{-/-}$	none	36/36	5	624.2 ± 133.30	0.89 ± 0.25	29.16 ± 1.93	26.93 ± 30.41***
Atp7b$^{-/-}$	100 μl water	36/44	5	592.6 ± 155.82	0.82 ± 0.29	26.4 ± 9.48	16.73 ± 8.41***
Atp7b$^{-/-}$	1 mg bc-TTM/kg bw	36/44	5	552.4 ± 263.0	0.78 ± 0.16	28.26 ± 11.02	10.91 ± 8.03
Atp7b$^{-/-}$	5 mg bc-TTM/kg bw	36/44	5	694.2 ± 268.58	0.81 ± 0.14	24.57 ± 8.15	16.83 ± 13.86
Atp7b$^{-/-}$	200 mg TETA/kg bw	36/44	6	461.67 ± 117.88	0.55 ± 0.08	21.94 ± 4.06	7.0 ± 3.88
Atp7b$^{-/-}$	200 mg DPA/kg bw	36/44	4	469.25 ± 76.71	0.64 ± 0.16	21.98 ± 4.92	6.47 ± 5.98

* Animals received oral gavages of indicated quantities of bis-choline-tetrathiomolybdate (bc-TTM), trientine (TETA), or D-penicillamine (DPA) dissolved in 100 μl water four times a week per oral gavage for 4 or 8 weeks. Animals receiving water only or sacrificed without any treatment served as controls. Data on hepatic copper of untreated *Atp7b*$^{-/-}$ mice were taken from previous measurements of our laboratory [22]. ** Values depicted indicate the spatial distribution of copper within the tissue. *** Hepatic Cu content in the *Atp7b*$^{-/-}$ mice at age 44 weeks that received water are lower than those of animals at age 36 weeks sacrificed at the beginning of the experiment, possibly reflecting burned out cirrhosis at later stages. Abbreviations used are: *Atp7b*$^{-/-}$, mouse deficient for the *Atp7b* gene; bw, body weight; weeks; WT, wild type.

Supplementary Figures

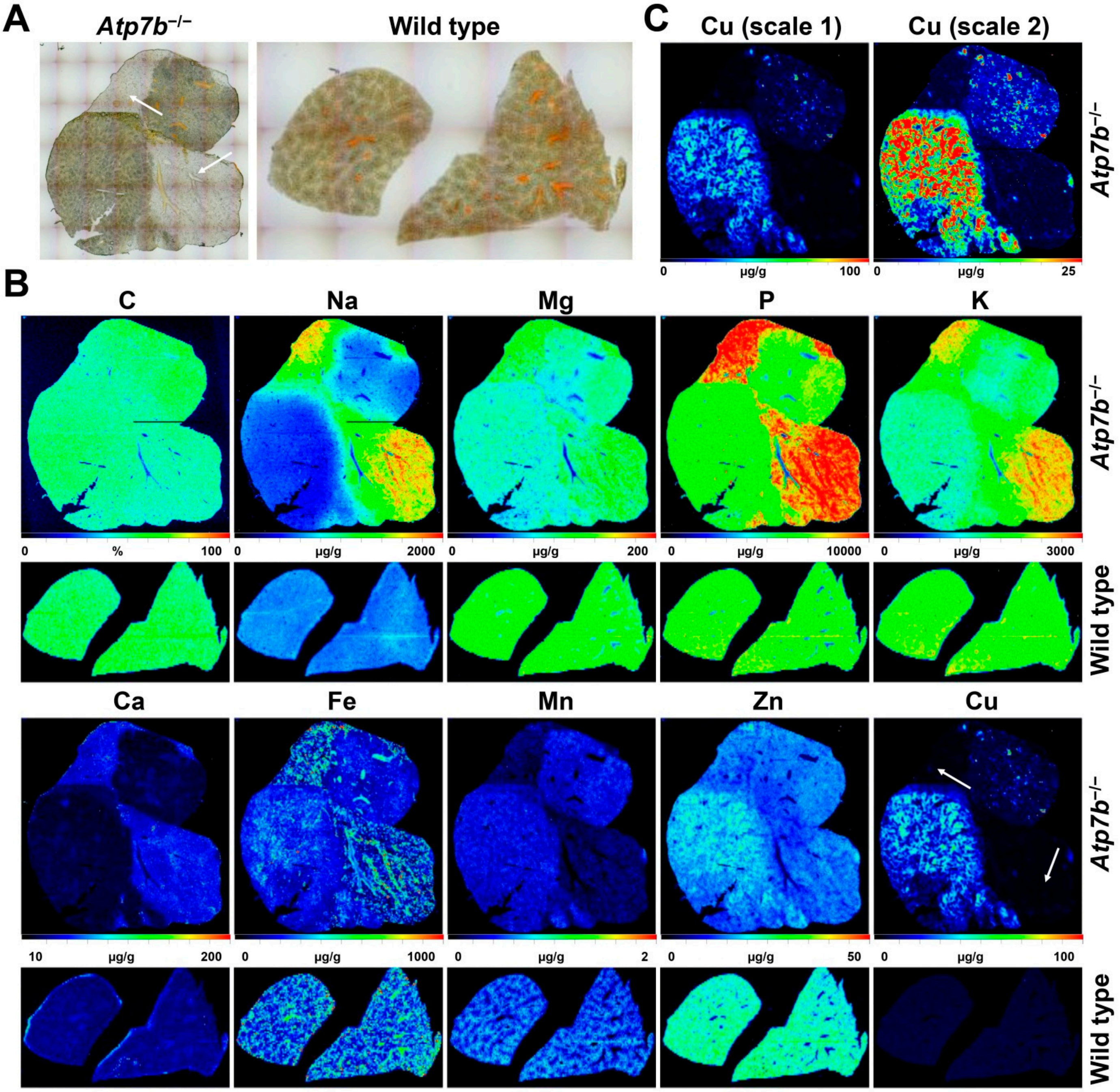

Figure S1. Analysis of the *Atp7b*$^{-/-}$ copper overload model by LA-ICP-MS. **(A)** Light microscopy of representative unstained liver sections from 10-month-old *Atp7b*$^{-/-}$ and wild type mice. Note the typical regenerative nodules frequently occurring in the *Atp7b*$^{-/-}$ mice. **(B)** Representative LA-ICP-MS images for carbon (C), sodium (Na), magnesium (Mg), phosphorus (P), potassium (K), calcium (Ca), iron (Fe), manganese (Mn), zinc (Zn), and copper (Cu) determined from liver sections shown in **(A).** Please note the lowered Cu content in the regenerative nodules that are marked by white arrows in (**A**) and (**B**). **(C)** Cu content visualized in different scales (i.e., 0-100 µg/g and 0-25 µg/g liver tissue) from the *Atp7b*$^{-/-}$ specimen depicted in **(B)**. Most favorable for image generation is a range in which the mean concentration of the measured element is in the middle (i.e., in this case the green range) of the scale. Under these conditions, the overload with Cu is best represented.

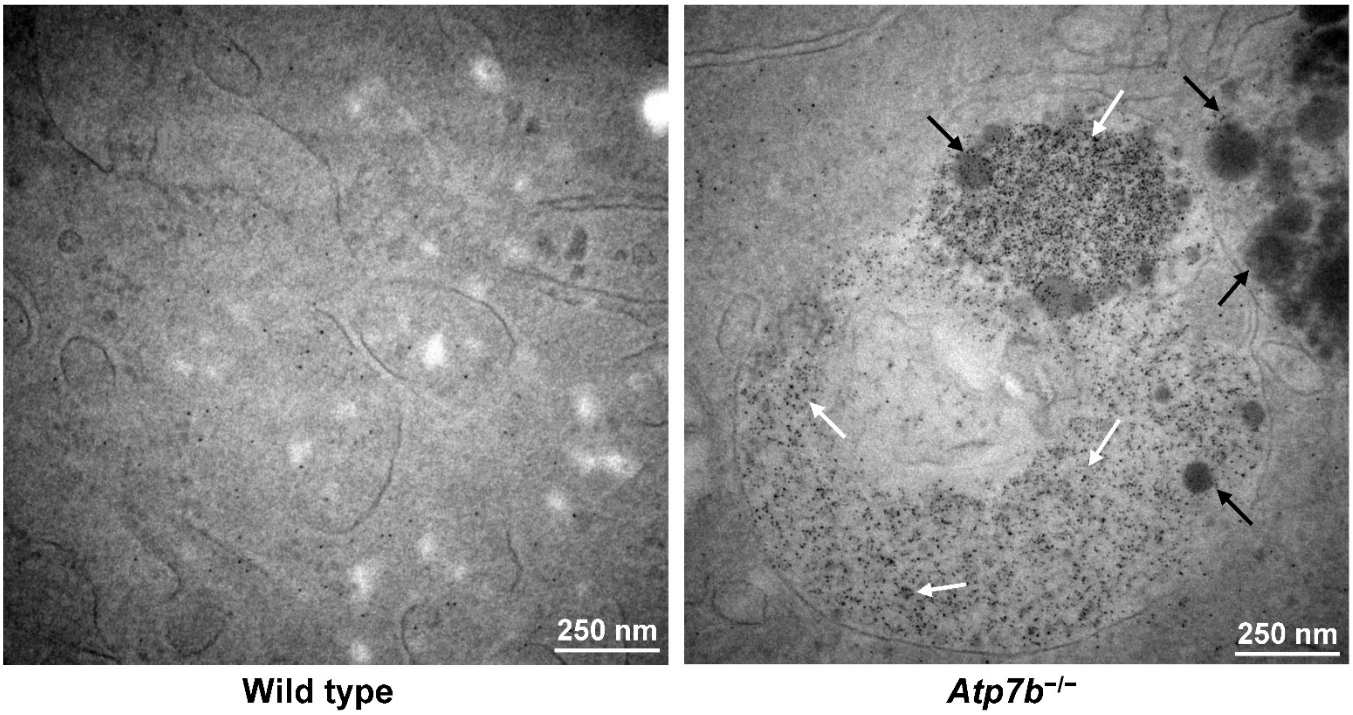

Figure S2. Electron microscopy of mouse liver specimens. Representative liver pieces from wild type (*left*) and *Atp7b*$^{-/-}$ mice (*right*) were fixed and examined by transmission electron microscopy. The *Atp7b*$^{-/-}$ samples showed frequent electron-dense lysosomal deposits (*white arrows*) and lipid droplets (*black arrows*) not observed in wild type samples. Space bars represent 250 nm. Abbreviations used are: *Atp7b*$^{-/-}$, mouse deficient for the *Atp7b* gene.

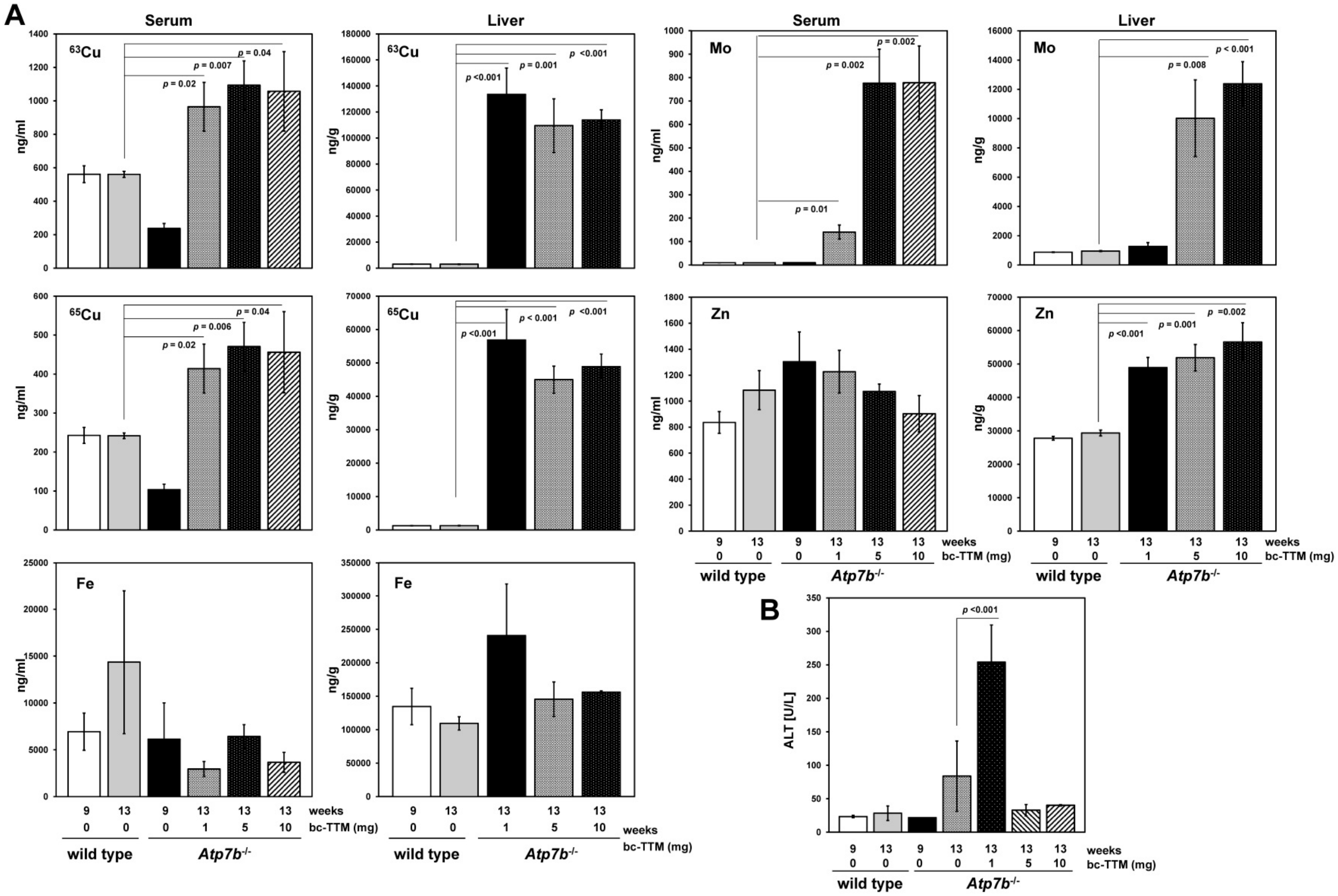

Figure S3. Element determination by inductively coupled plasma mass spectrometry (ICP-MS) in serum and liver samples and analysis of alanine aminotransferase (ALT). **(A)** ^{63}Cu, ^{65}Cu, Fe, Mo and Zn were determined in serum in ashed liver samples by ICP-MS (each n=3). For individual values determined in each animal refer to Suppl. Table 2 **(B)** ALT measurements of serum samples isolated from animals of the respective groups. Values of significance are given. Abbreviations used are: *Atp7b*$^{-/-}$, mouse deficient for the *Atp7b* gene; bc-TTM, bis-choline-tetrathiomolybdate; DPA, D-penicillamine; TETA, trientine.

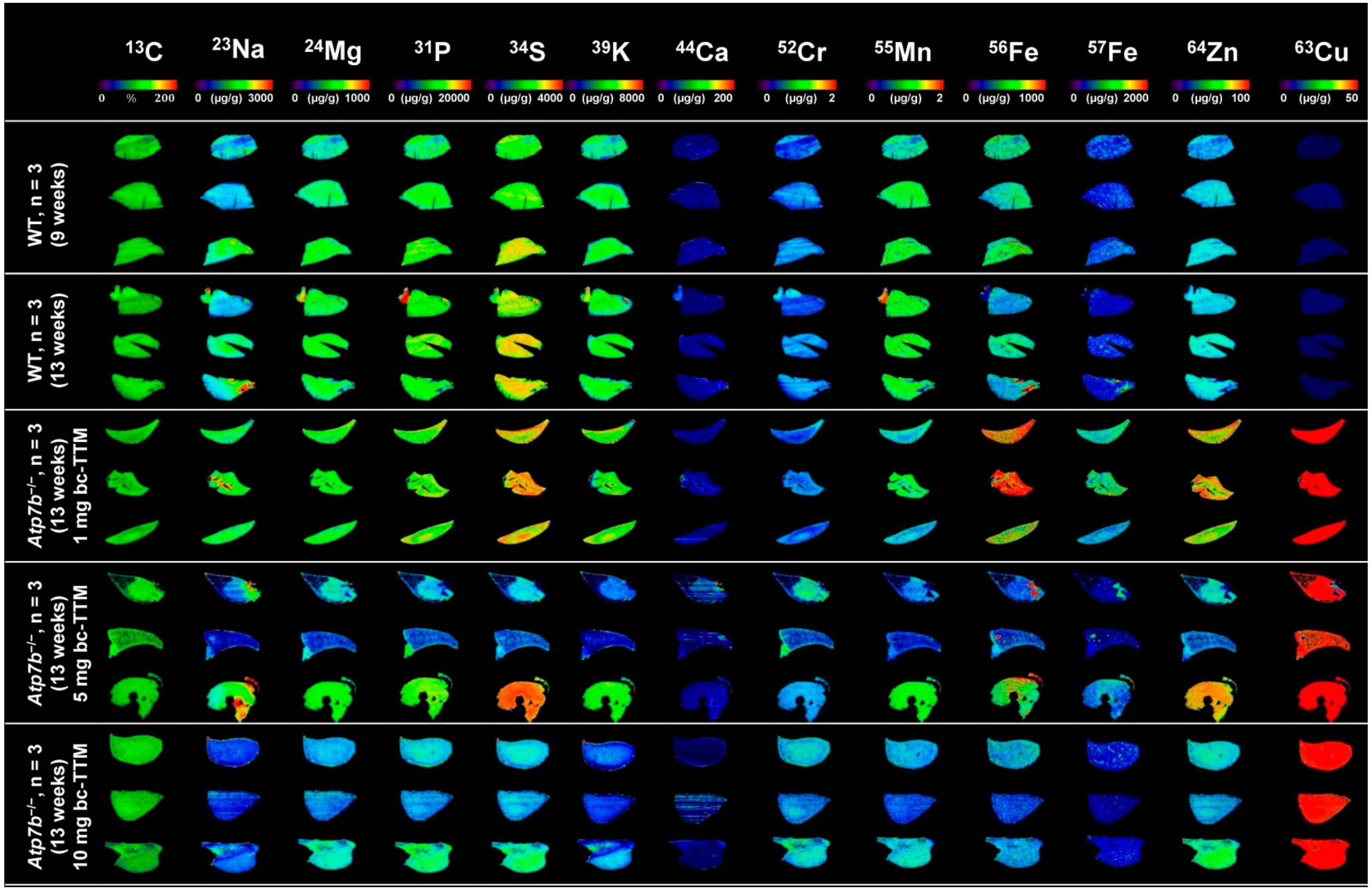

Figure S4. LA-ICP-MS imaging in liver sections of *Atp7b*$^{-/-}$ mice treated with different quantities of bis-choline-tetrathiomolybdate (bc-TTM). Simultaneous LA-ICP-MS imaging of carbon (^{13}C), sodium (^{23}Na), magnesium (^{24}Mg), phosphorus (^{31}P), sulfur (^{34}S), potassium (^{39}K), calcium (^{44}Ca), chrome (^{52}Cr), manganese (^{55}Mn), iron (^{56}Fe, ^{57}Fe), copper (^{63}Cu), and zinc (^{64}Zn) was done in cryo-cuts from *Atp7b*$^{-/-}$ mice treated with increasing concentrations (1, 5, and 10 mg/kg body weight, 3 animals per group) of bc-TTM. Specimens from normal wild type (WT) controls were measured in parallel to estimate the physiological concentrations of hepatic elements. Please note the correlation of elevated hepatic Cu concentrations and increased Fe and Zn content in the *Atp7b*$^{-/-}$ animals. In this overview, Cu is depicted in a scale from 0 to 50 µg/g liver tissue. Abbreviations used are: *Atp7b*$^{-/-}$, mouse deficient for the *Atp7b* gene; bc-TTM, bis-choline-tetrathiomolybdate; WT, wild type.

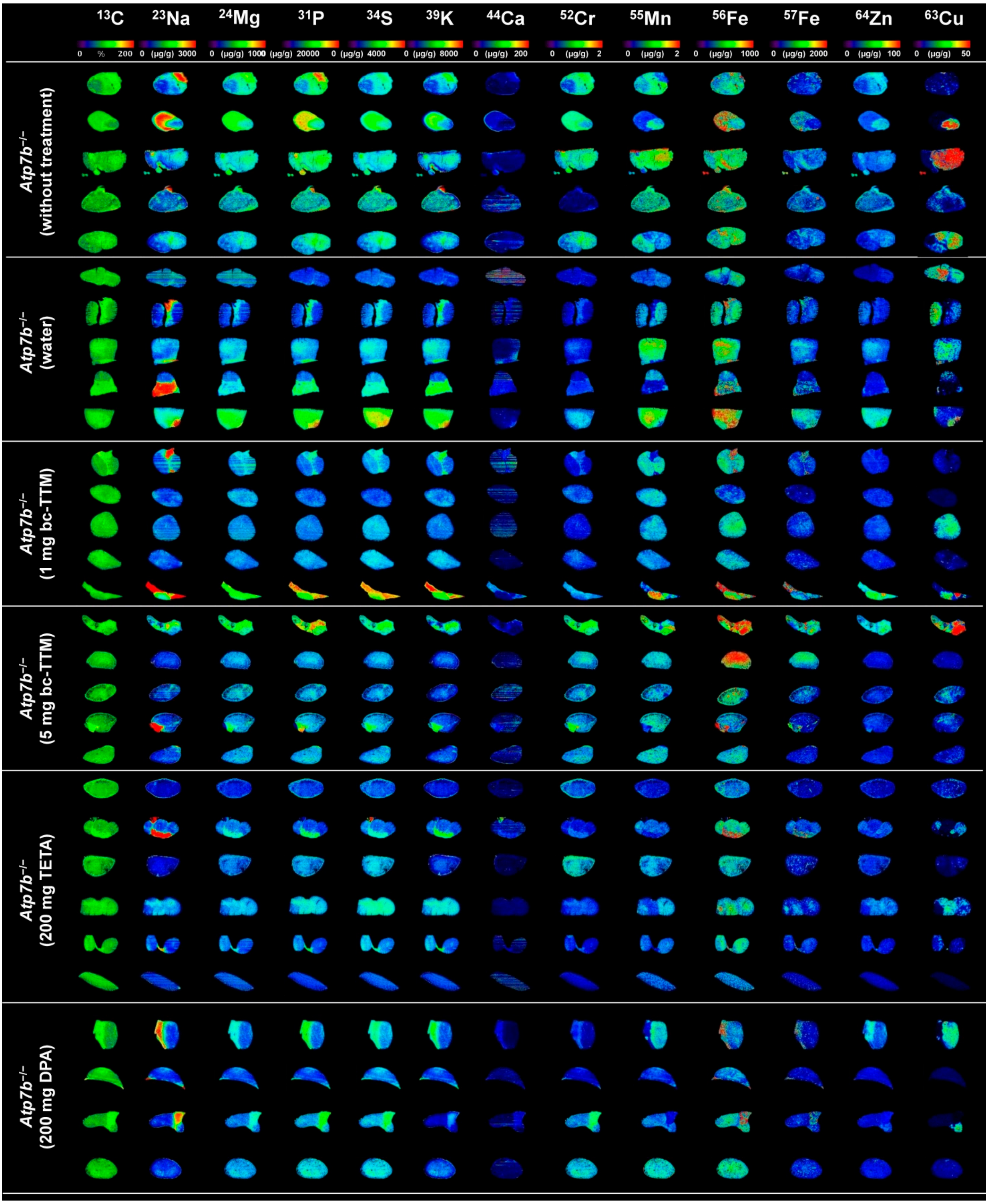

Figure S5. LA-ICP-MS imaging in liver sections of *Atp7b*$^{-/-}$ mice treated with different chelators. Simultaneous LA-ICP-MS imaging of carbon (^{13}C), sodium (^{23}Na), magnesium (^{24}Mg), phosphorus (^{31}P), sulfur (^{34}S), potassium (^{39}K), calcium (^{44}Ca), chrome (^{52}Cr), manganese (^{55}Mn), iron (^{56}Fe, ^{57}Fe), copper (^{63}Cu), and zinc (^{64}Zn) was done in liver sections of the different treatment and control groups. Please note the inhomogeneous element distribution in the untreated and water control groups that is majorly due to formation of regenerative nodules. Abbreviations used are: *Atp7b*$^{-/-}$, mouse deficient for the *Atp7b* gene; bc-TTM, bis-choline-tetrathiomolybdate; DPA, D-penicillamine; TETA, trientine.

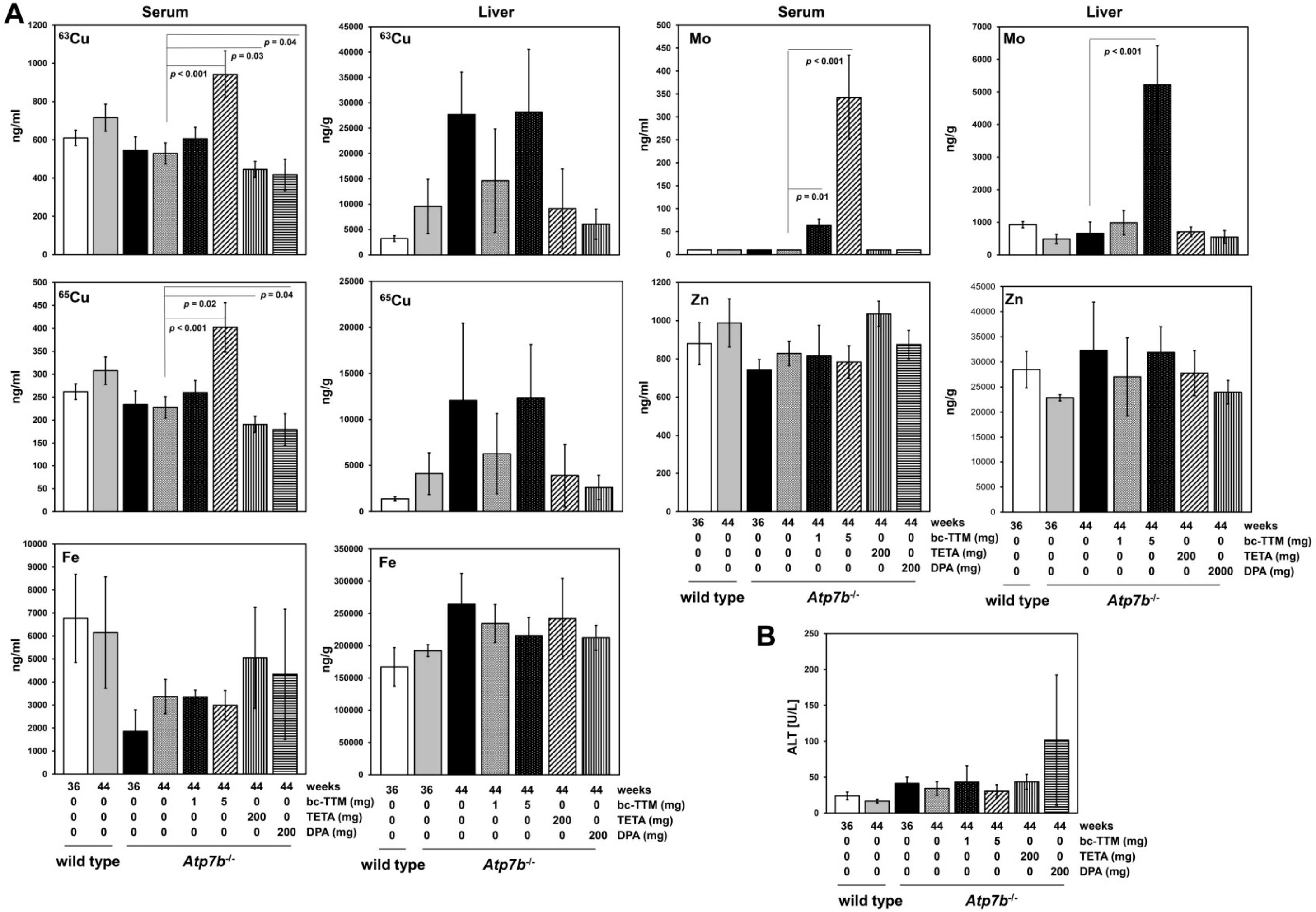

Figure S6. Element determination by inductively coupled plasma mass spectrometry (ICP-MS) in serum and liver samples and analysis of alanine aminotransferase (ALT). **(A)** ^{63}Cu, ^{65}Cu, Fe, Mo and Zn were determined in serum in ashed liver samples by ICP-MS (each n=5-8 in controls and bc-TTM groups, n=6 in TETA and DPA groups. For individual values determined in each animal refer to Suppl. Table 2. **(B)** ALT measurements of serum samples isolated from animals of the respective groups. Values of significance are given. Abbreviations used are: *Atp7b*$^{-/-}$, mouse deficient for the *Atp7b* gene; bc-TTM, bis-choline-tetrathiomolybdate; DPA, D-penicillamine; TETA, trientine.
